# Pathways to empowerment: integrating psychoeducation, peer support, and co-production for lasting recovery

**DOI:** 10.1192/j.eurpsy.2026.10295

**Published:** 2026-07-31

**Authors:** P. Neckar, N. Boxall

**Affiliations:** Forensic, Oxford Health NHS Foundation Trust, Oxford, United Kingdom

## Abstract

**Abstract:**

In the United Kingdom, forensic mental health services are funded under the National Health Service (NHS) and provide care in secure settings for individuals with complex mental health needs and involvement with the criminal justice system. While some services are delivered by private providers, this presentation focuses on NHS provision at Oxford Health NHS Foundation Trust.

Forensic services can feel highly supportive but are also risk-focused and restrictive. Unlike prison, where timeframes are often clearer, detention within forensic mental health services does not usually have a fixed end point, making it difficult for individuals to plan for the future and sustain progress. Extended stays within secure environments can contribute to institutionalisation effects, and individuals may experience dual stigma: stigma associated with mental ill health alongside stigma linked to past offending. Together, these factors can significantly impact identity, agency, and hope.

In response, empowerment is positioned as a core principle of recovery-oriented practice. A key component is the integration of peer support workers—individuals with lived experience of mental health services—who are employed to use that experience in a formal and recovery-focused way. Peer support within forensic settings is grounded in mutuality and relationship-building rather than clinical intervention, and can help challenge stigma, soften “us and them” dynamics, and strengthen personal agency.

Peer support is closely aligned with the Recovery College model, and this interrelationship is explored. Recovery Colleges provide structured educational opportunities for people detained within forensic mental health services, with opportunities to rebuild identities beyond illness- and offence-based narratives. Central is a progressive pathway of participation and empowerment, through which individuals may move from patient to student, from student to Expert by Experience tutor, and in some cases from Expert by Experience to active stakeholder within Recovery College leadership and development.

Recovery Colleges offer annual programmes of co-produced psychoeducational and recovery-focused courses that support individuals to develop skills, increase self-understanding, and consolidate recovery-oriented identities. Collaboration sits at the core of this approach, with service users, peer support workers, mental health professionals, and creative practitioners working together as equal partners in learning, delivery, and shared experience. This presentation reflects on how integrating peer support and Recovery College approaches within forensic mental health services can promote empowerment, shared ownership, and recovery-focused cultures within secure care.

**Disclosure of Interest:**

P. Neckar Employee of: Employee of: Employee of Oxford Health NHS Foundation trust, N. Boxall Employee of: Employee of Oxford Health NHS Foundation trust

